# Integrating Ultrasound-Guided Injections and Peripheral Magnetic Stimulation in Chronic Myofascial/Lumbar Pain

**DOI:** 10.3390/life15040563

**Published:** 2025-03-31

**Authors:** Wei-Ting Wu, Ke-Vin Chang, Kamal Mezian, Vincenzo Ricci, Levent Özçakar

**Affiliations:** 1 Department of Physical Medicine and Rehabilitation, National Taiwan University Hospital, Bei-Hu Branch, Taipei 10845, Taiwan; b40560@bh.ntuh.gov.tw; 2 Department of Physical Medicine and Rehabilitation, National Taiwan University College of Medicine, Taipei 100233, Taiwan; 3 Center for Regional Anesthesia and Pain Medicine, Wang-Fang Hospital, Taipei Medical University, Taipei 110301, Taiwan; 4 Department of Rehabilitation Medicine, Charles University, First Faculty of Medicine and General University Hospital in Prague, 12800 Prague, Czech Republic; kamal.mezian@lf1.cuni.cz; 5 Physical and Rehabilitation Medicine Unit, Luigi Sacco University Hospital, ASST Fatebenefratelli-Sacco, 20133 Milan, Italy; ricci.vincenzo@asst-fbf-sacco.it; 6 Department of Physical and Rehabilitation Medicine, Hacettepe University Medical School, Ankara 06100, Turkey; lozcakar@hacettepe.edu.tr

**Keywords:** muscle, painful, ultrasonography, intervention, multifidus

## Abstract

Myofascial pain syndrome (MPS) is a common musculoskeletal disorder that significantly affects quality of life. Conventional treatment approaches include pharmacological interventions, physical therapy, and procedures such as dry needling. Among these, ultrasound-guided injections (USGIs) have gained recognition for their precision and therapeutic benefits. Additionally, repetitive peripheral magnetic stimulation (rPMS) has emerged as a non-invasive neuromodulatory technique for pain management. This perspective article examines the physiological mechanisms and clinical applications of USGIs and rPMS, particularly in the lumbar multifidus muscle, and explores their potential synergistic effects. MPS is often associated with chronic muscle dysfunction due to energy depletion, leading to persistent pain and motor impairment. USGIs play a crucial role in restoring muscle perfusion, disrupting pain cycles, and providing diagnostic insights in real time. In parallel, rPMS modulates neuromuscular activation, enhances endogenous pain control, and promotes functional recovery. Ultrasound guidance enhances the precision and effectiveness of interventions, such as dry needling, interfascial plane blocks, and fascial hydrodissection, while rPMS complements these strategies by facilitating neuromuscular reconditioning and reducing pain via central and peripheral mechanisms. The preliminary findings suggest that combining multifidus USGIs with rPMS results in significant pain relief and functional improvements in patients with chronic low back pain. Integrating USGIs with rPMS represents a promising multimodal strategy for managing MPS. By combining targeted injections with non-invasive neuromodulation, clinicians may optimize therapeutic outcomes and provide sustained relief for patients with chronic musculoskeletal pain. Further research is needed to refine treatment protocols and assess the long-term efficacy.

## 1. Introduction

Myofascial pain syndrome (MPS) is a prevalent musculoskeletal condition, affecting a significant portion of the general population. Epidemiological studies suggest that it accounts for a substantial percentage of chronic pain disorders, with estimates indicating a prevalence of up to 85% in patients presenting with musculoskeletal complaints [1]. It is particularly common in individuals with sedentary lifestyles, repetitive strain injuries, or underlying musculoskeletal disorders. If untreated, MPS can lead to persistent discomfort, functional impairment, and a reduced quality of life [2]. The chronic nature of the condition increases the risk of central sensitization, contributing to widespread pain syndromes and complicating clinical management [3].

Conventional treatment strategies for MPS include medical treatment (e.g., nonsteroidal anti-inflammatory drugs (NSAIDs), muscle relaxants), physical therapy (acupuncture, massage, transcutaneous electrical stimulation, and interferential current therapy), and various interventions (e.g., dry needling) [4,5]. Among these approaches, ultrasound-guided injections (USGIs) have gained increasing recognition for their ability to enhance precision, optimize therapeutic delivery, and minimize procedural complications [6]. Various techniques, such as dry needling, interfascial plane blocks, and fascial hydrodissection [6,7], have been utilized to target myofascial trigger points (MTrPs) and surrounding fascial layers. Dry needling, when performed under ultrasound guidance, allows for the direct visualization of the needle within the affected muscle, with a simultaneous twitch response [7]. Interfascial plane blocks facilitate the delivery of anesthetic agents into specific fascial compartments, providing effective pain relief in patients with deeply-seated myofascial pain [7]. Similarly, fascial hydrodissection—using fluid injection to separate adhered fascial layers—has shown promise in restoring tissue mobility and alleviating myofascial restrictions [7].

Beyond injection-based therapies, repetitive peripheral magnetic stimulation (rPMS) has also emerged as a non-invasive adjunct for managing MPS [8]. rPMS generates electromagnetic fields that induce painless neuromuscular stimulation, promoting local circulation, muscle relaxation, and pain modulation. Notably, combining rPMS with USGIs may offer synergistic benefits, as targeted injections can first disrupt pain generators within the myofascial structures, followed by rPMS to enhance neuromuscular reconditioning. This multimodal approach holds significant potential for optimizing pain relief and functional recovery in patients with chronic MPS.

This perspective article examines the physiological mechanisms, clinical applications, and supporting evidence for combined USGIs and rPMS in managing MPS. By integrating these emerging techniques, clinicians may enhance treatment efficacy and achieve more sustainable pain relief for individuals with chronic musculoskeletal conditions.

## 2. Pathophysiology of Myofascial Pain

MPS is characterized by hypersensitive areas within muscles, known as MTrPs. They develop due to excessive and sustained contraction of sarcomeres, the fundamental contractile units of muscle fibers. According to the sliding filament theory, muscle contraction is driven by the interaction between thick (myosin) and thin (actin) filaments [9]. Myosin heads cyclically attach to actin, forming cross-bridges that generate force through power strokes, a process dependent on adenosine triphosphate (ATP). ATP not only fuels contraction but is also essential for muscle relaxation, as it allows myosin heads to detach from actin and reset for subsequent contractions.

In MPS, chronic overactivation of sarcomeres leads to a persistent contracture state at the level of the affected muscle fibers [10]. This sustained contraction results in localized ischemia and hypoxia, impairing ATP production. As ATP depletion prevents myosin detachment from actin, muscle fibers remain in a rigid, shortened state, much like the mechanism seen in rigor mortis. This prolonged contracture disrupts normal muscle function and initiates a cascade of pathological changes, including an increased metabolic demand, impaired local circulation, and the accumulation of nociceptive mediators such as bradykinin, substance P, and pro-inflammatory cytokines [11]. Clinically, these biochemical alterations manifest as hyperalgesia, allodynia, muscle stiffness, and restricted range of motion.

This energy deficiency model extends beyond MPS to other musculoskeletal conditions [11], such as dead buttock syndrome, in which chronic ATP depletion contributes to gluteal muscle atrophy and pain. It may also be relevant to muscle degeneration processes if neuropathy is excluded. In such cases, prolonged disuse or dysfunction of the gluteal muscles leads to metabolic inefficiency, impairing their ability to generate and utilize ATP effectively. The resulting muscular fatigue, weakness, and myofascial dysfunction further perpetuate pain and movement restrictions, creating a cycle of chronic discomfort and functional decline.

Recent studies have shed light on the molecular pathways underlying MTrPs. For example, Liu et al. [12] identified PDGFR-α activation as a key factor in MTrP development. Its elevated phosphorylation correlates with pain intensity and drives muscle contraction and pain-like behaviors via the JAK2/STAT3 pathway, while the inhibition of this pathway reverses these effects. Additionally, COL1A1 enhances PDGFR-α phosphorylation, contributing to MTrP-associated pain and dysfunction.

## 3. Effects of Ultrasound-Guided Injections

**Figure 1:**
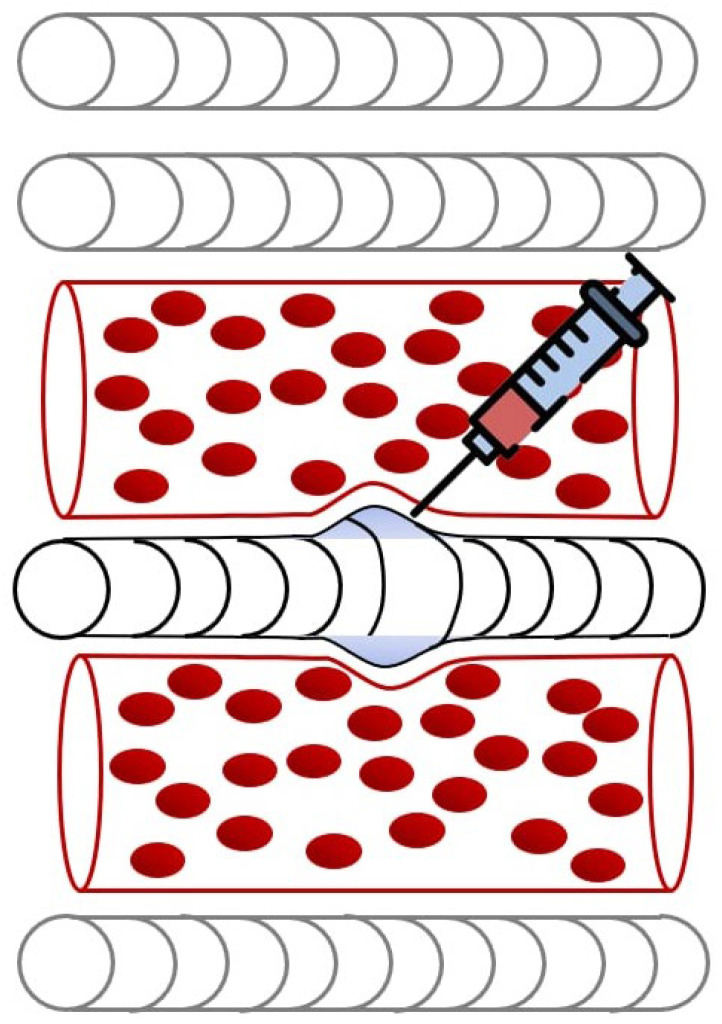
Prolonged sarcomere contraction impairs blood flow, which may be alleviated by ultrasound-guided needling.

One of the primary advantages of USGIs is their ability to precisely target dysfunctional muscle regions, optimizing therapeutic outcomes while minimizing risks associated with blind techniques. USGIs play a critical role in breaking this pathological cycle by promoting angiogenesis and improving local perfusion (Figure 1). By restoring oxygen delivery to ischemic tissues, these injections alleviate metabolic stress, facilitating muscle relaxation and reducing pain. This mechanism is evident in the management of MPS of quadratus lumborum (QL), a common source of chronic low back pain [13]. A recent observational and retrospective study [13] assessed the efficacy of USGI of the QL muscle with 0.25% levobupivacaine and 40 mg triamcinolone in 90 participants over a four-year period. The study found significant pain reduction at multiple time points post-intervention, with improvements observed at 72 h (mean difference [MD] = 3.085, 95% CI: 2.200–3.970, *p* < 0.05), 1 month (MD = 2.644, 95% CI: 1.667–3.621, *p* < 0.05), 3 months (MD = 2.017, 95% CI: 0.202–2.729, *p* < 0.05), and also at 6 months (MD = 1.339, 95% CI: 0.378–2.300, *p* < 0.05). Notably, the intervention did not lead to increased opioid consumption or adverse effects, reinforcing the safety and long-term efficacy of the technique.

Beyond pain relief, USGIs provide valuable diagnostic insights by assessing real-time muscle response to injection. The nature of the muscle’s reaction can indicate its physiological state, as a slow-twitch response suggests chronic energy depletion and muscle fatigue, while a rapid-twitch response may indicate preserved neuromuscular function and recovery potential, which can be explained by the impairment of excitation–contraction coupling due to muscle fatigue, leading to reduced twitch tension and prolonged recovery times [14]. Notably, this real-time feedback allows clinicians to tailor treatment plans, integrating additional interventions such as interfascial plane blocks and fascial hydrodissection for more comprehensive pain management [7].

## 4. Multifidus Muscle and Low Back Pain

**Figure 2:**
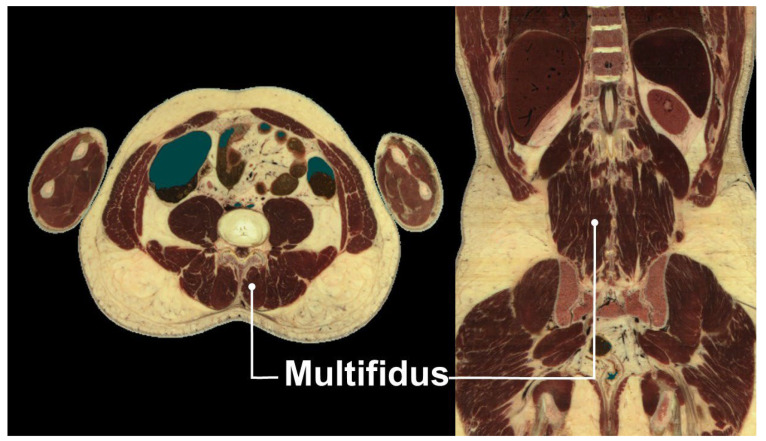
Cadaveric transverse and coronal cross-sectional images showing the multifidus muscle. Images adapted from cadaveric images provided by the Visible Human Project^®^ of the National Library of Medicine. Excerpts featured in the VH Dissector are used with permission from Touch of Life Technologies Inc.

The multifidus muscle, a key stabilizer of the lumbar spine, is highly susceptible to atrophy in patients with chronic low back pain [15]. This deep paraspinal muscle is part of the transversospinalis group and consists of short, segmental fibers spanning two to five vertebral levels. Originating from the sacrum, mammillary processes of the lumbar vertebrae, and transverse processes of the thoracic vertebrae, it inserts onto the spinous processes of superior vertebrae (Figure 2). Its unique anatomical arrangement provides segmental stabilization, counteracting shear forces and maintaining spinal alignment.

As essential stabilizers of the “neutral zone” range of motion, lumbar multifidus muscles play a critical role in spinal biomechanics. T2-weighted magnetic resonance imaging without fat suppression shows that high-intensity signals occupying more than 50% of the muscle’s cross-sectional area correlate with significant atrophy and fatty infiltration, indicating persistent dysfunction [16]. This degenerative process compromises spinal stability and underscores the importance of targeted rehabilitation strategies.

## 5. Ultrasound-Guided Injections Targeting the Multifidus Muscle

**Figure 3:**
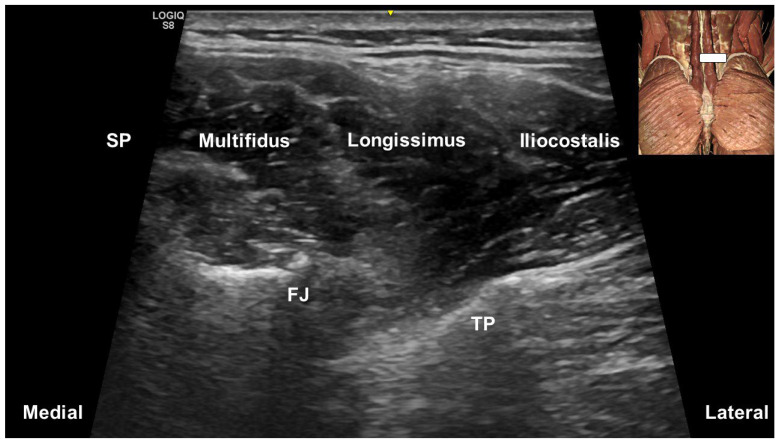
Ultrasound imaging of multifidus and other paraspinal muscles in the axial plane. FJ, facet joint; TP, transverse process; SP, spinous process.

In their pictorial review, Hung et al. [17] described the lumbar multifidus as originating from the mammillary processes of the lumbar vertebrae and the posterior surface of the sacrum (Figure 3). At the upper lumbar levels, the multifidus is confined to the region over the laminae, gradually expanding to cover the sacral bone as the transducer moves caudally from the L3 level. Laterally, the longissimus thoracis and iliocostalis lumborum converge onto the transverse process.

Suputtitada et al. [18] elaborated on USGIs with 5% dextrose into the multifidus muscle for treating myofascial pain. Dextrose is believed to promote local tissue repair and reduce nociceptive input. Their study investigated the effectiveness of three USGI methods in elderly patients with lumbar facet joint syndrome, a common cause of lower back pain. Sixty patients were divided into three groups receiving either medial branch block, facet joint injection, or injections into the multifidus muscle. All groups were treated with 5% dextrose water, and pain severity was assessed using the visual analog scale (VAS). Before treatment, the average VAS score was approximately 7.5. After three consecutive injections at two-week intervals, the three groups had similar results, whereby the scores significantly decreased to around 1 (mild/no pain). Given its relative ease of administration compared to medial branch block and facet injection, USGIs of the multifidus may be a feasible treatment option in elderly patients with facet joint-related low back pain.

**Figure 4:**
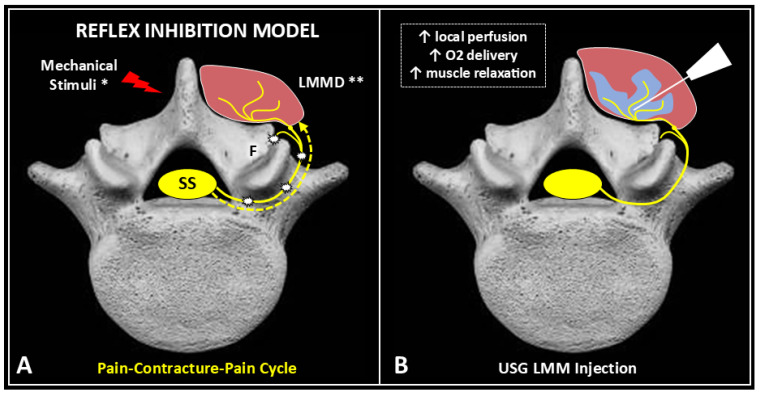
Mechanical stimuli (red flash) activate an inhibitory spinal reflex (**A**). Hyperactivation of intramuscular proprioceptors of the multifidus muscle (brown) generates continuous nociceptive afferent feedback (white flashes) via the dorsal ramus of the spinal nerve (yellow line), leading to efferent inhibitory signals (dotted curved yellow arrow) to the muscle fibers. Lumbar multifidus muscle dysfunction (LMMD) causes spinal sensitization (SS) as persistent contracture sustains afferent pain signals to the posterior horn of the spinal cord (yellow dot). Ultrasound-guided injection of the lumbar multifidus muscle (**B**) reactivates muscle fibers, counteracting spinal sensitization and interrupting the inhibitory reflex. F: facet; *: chronic overload of the vertebral segment; **: chronic myofascial pain; light blue: injectate; white: needle.

The effectiveness of lumbar multifidus injection for lumbar myofascial pain may be explained through the modulation of the reflex inhibition model [16,19,20]. Excessive mechanical stimuli on the lumbar spine activate an inhibitory spinal reflex. Hyperactivation of the multifidus muscle’s intramuscular proprioceptors causes continuous nociceptive afferent feedback via the dorsal ramus of the spinal nerve, followed by efferent inhibitory signals to the muscle fibers (Figure 4A). Lumbar multifidus muscle dysfunction results in progressive spinal sensitization, as persistent muscle contracture generates continuous afferent pain signals to the posterior horn of the spinal cord. An ultrasound-guided injection into the lumbar multifidus muscle reactivates muscle fibers and interrupts spinal sensitization, counteracting the inhibitory reflex (Figure 4B).

## 6. Repetitive Peripheral Magnetic Stimulation

### 6.1. Fundamental Principles

rPMS is a non-invasive neuromodulation technique based on Faraday’s law of electromagnetic induction [21], which states that a changing magnetic field induces an electric current in a nearby conductor. In rPMS, a rapidly oscillating magnetic field is generated by passing an electric current through a coil, leading to the induction of electric currents within biological tissues. These currents depolarize neurons, resulting in neuromuscular stimulation. Unlike transcutaneous electrical nerve stimulation (TENS), which delivers electrical currents directly through the skin, rPMS bypasses the high impedance of superficial tissues, enabling deeper penetration with minimal discomfort. This allows for the activation of motor and sensory nerves at a greater depth, making it particularly useful in treating musculoskeletal and neurological disorders [8].

### 6.2. Mechanisms of Pain Relief

The analgesic effects of rPMS are partially explained by the gate control theory [22], which suggests that stimulation of A-beta fibers (fast-conducting, non-nociceptive sensory fibers) inhibits the transmission of pain signals from slower-conducting C-fibers in the spinal cord. This process reduces pain perception and contributes to immediate symptom relief.

**Figure 5:**
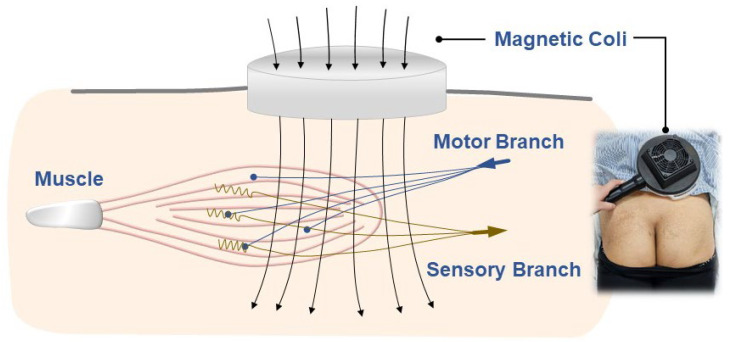
Illustration depicting the effects of repetitive peripheral magnetic stimulation on sensory modulation and motor recruitment in low back muscles.

Beyond sensory modulation, rPMS promotes motor unit recruitment, enhancing muscle activation and functional recovery (Figure 5). This effect has been observed in conditions, such as musculoskeletal pain syndromes [23], where improved muscle strength and neuromuscular coordination are important for rehabilitation. Additionally, rPMS may facilitate endogenous pain modulation mechanisms, including the release of endorphins and the activation of descending inhibitory pathways.

### 6.3. Machine Components

**Figure 6:**
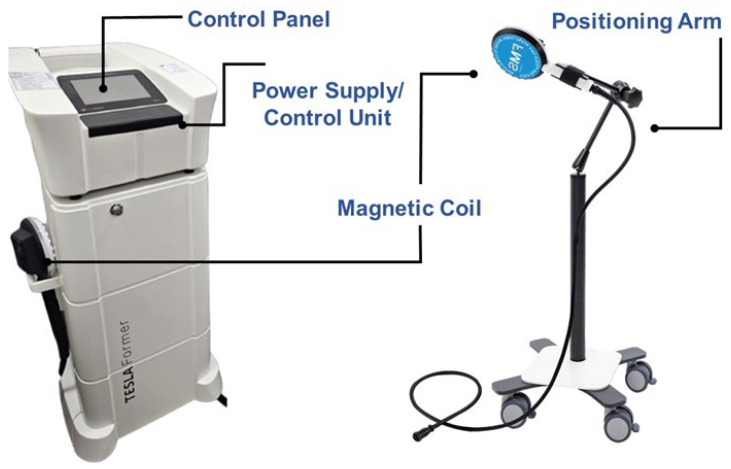
Illustration of the main components of a repetitive peripheral magnetic stimulation machine. This illustration is adapted from photos taken at the corresponding author’s hospital, which is equipped with a TESLA Stym^®^ device (Iskra Medical d.o.o., Podnart, Slovenia).

An rPMS machine consists of several key components (Figure 6). The power supply unit provides the necessary electrical energy to generate high-intensity, rapidly alternating currents. The control unit regulates the frequency, intensity, and duration of stimulation based on therapeutic protocols. The magnetic coil (stimulation applicator) converts electrical energy into a time-varying magnetic field, typically made of insulated copper wire wound into a specific geometry to optimize field strength and focus. Since prolonged use can generate heat, a cooling system, either air- or liquid-based, prevents overheating and maintains performance. The user interface, often a touchscreen or control panel, allows clinicians to adjust stimulation parameters, ensuring personalized treatment. A positioning arm or holder secures the coil at the desired treatment location, ensuring consistency in application.

### 6.4. Comparison with Transcutaneous Electrical Nerve Stimulation

**Figure 7:**
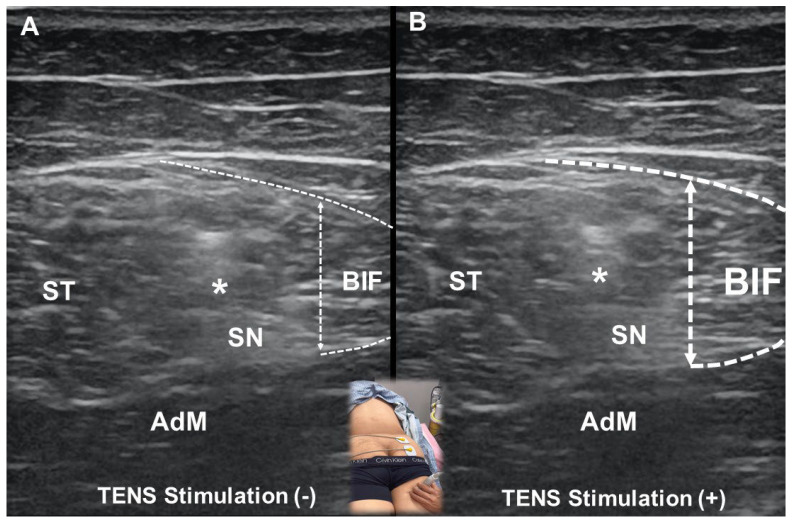
Placement of the electrode over the buttock for high-frequency transcutaneous electrical nerve stimulation (TENS) at baseline (**A**) and during stimulation (**B**) when focal contraction of the biceps femoris (BIF) is observed with increased thickness. ST, semitendinosus; SN, sciatic nerve; AdM, adductor magnus; *, conjoint tendon.

While both rPMS and TENS are non-invasive modalities used for pain management, they differ in terms of mechanisms, depth of penetration, patient comfort, and therapeutic applications. TENS primarily functions through electrical stimulation applied via surface electrodes to modulate pain perception. It activates large-diameter afferent fibers, which inhibit pain transmission at the spinal level through the gate control theory. Additionally, high-frequency TENS (≥50 Hz) is thought to induce analgesia by stimulating endogenous opioid release, particularly activating δ-opioid receptors [24], whereas low-frequency TENS (<10 Hz) targets μ-opioid receptors [25]. In contrast, rPMS generates electromagnetic fields that penetrate tissues to depolarize neuromuscular structures, facilitating deep muscle activation and neuromodulation [26]. Unlike TENS, which predominantly affects sensory nerves, rPMS can elicit both sensory and motor responses, leading to muscle contractions that promote functional recovery. Due to its reliance on electrical currents, TENS is limited to superficial nerve stimulation [27], making it less effective for targeting deep-seated musculoskeletal structures (Figure 7 and Video S1).

**Figure 8:**
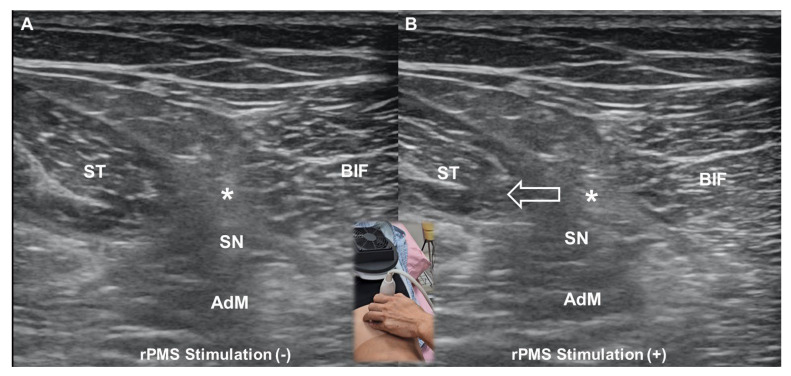
Placement of the applicator over the buttock for repetitive peripheral magnetic stimulation (rPMS) at baseline (**A**) and during stimulation (**B**) when the entire posterior thigh musculature contracts, causing the conjoint tendon of the hamstrings to shift medially (void arrow) while the hip and knee joints remain fixed. ST, semitendinosus; SN, sciatic nerve; AdM, adductor magnus; BIF, biceps femoris; *, conjoint tendon.

Conversely, rPMS induces electric fields within tissues without requiring direct skin contact. This allows for the stimulation of deeper muscles and nerves, which is particularly beneficial for neuromuscular re-education and pain modulation in conditions involving deeper structures (Figure 8 and Video S2). While TENS is widely used for pain relief, its effectiveness is often constrained by patient tolerance. Higher intensities, which may be necessary for optimal analgesic effects, frequently cause discomfort or even skin irritation. In contrast, rPMS delivers pulsed magnetic fields that generate electrical currents in tissues without direct skin stimulation, reducing the likelihood of discomfort while still achieving therapeutic neuromuscular effects.

### 6.5. Optimizing Parameters

A meta-analysis by Diao et al. [28] examined the effects of rPMS on pain intensity, functional mobility, and kinesiophobia in individuals with low back pain. Analyzing six randomized controlled trials involving 139 participants, they found that rPMS significantly reduced pain intensity (MD, −1.89; 95% CI, −3.32 to −0.47; *p* < 0.05) and improved functional disability (Oswestry Disability Index: MD, −8.39; 95% CI, −13.65 to −3.12; *p* < 0.001). However, it did not produce a significant between-group difference in kinesiophobia (MD, −1.81; 95% CI, −7.60 to 3.98; *p* > 0.05), suggesting that while rPMS effectively alleviates pain and disability, its influence on the psychological fear of movement remains unclear. Among the six included studies, frequencies between 10 and 50 Hz were optimal for musculoskeletal conditions, with 20 Hz being the most commonly used. Intensity levels typically ranged from 20% to 60%, with 45% as a standard setting for low back pain.

The effects of rPMS vary depending on the frequency used. Low-frequency rPMS (e.g., 5 Hz) has been shown to reduce the tendon reflex amplitude, which may contribute to pain relief and improved mobility. Zschorlich et al. [29] conducted a study in which 38 participants underwent either rPMS or sham stimulation applied to the posterior tibial nerve. They found that a single session of 5 Hz rPMS significantly reduced the soleus tendon reflex response by 23.7% (*p* < 0.001) compared to baseline, whereas no significant effects were observed in the sham group. This finding suggests that low-frequency rPMS can decrease muscle hypertonia and spasticity, potentially serving as an adjuvant therapy for neurorehabilitation in patients with impaired mobility.

Conversely, high-frequency rPMS (HF-rPMS) has been shown to enhance motor function and muscle strength, making it particularly suitable for rehabilitation. Ke et al. [30] conducted a randomized, double-blind, sham-controlled trial in patients with intracerebral hemorrhage, applying synchronous HF-rPMS to the axilla (targeting the brachial plexus) and the popliteal fossa (stimulating the tibial and common peroneal nerves). Among 26 patients who completed the study, HF-rPMS significantly improved the upper extremity (*p* = 0.012) and lower extremity (*p* = 0.001) FMA scores, proximal muscle strength of the upper limb (*p* = 0.043), and proximal (*p* = 0.004) as well as distal (*p* = 0.008) muscle strength of the lower limb. These results imply the potential of HF-rPMS in facilitating motor recovery in the acute and early subacute phases of stroke rehabilitation.

Taken together, these findings highlight the frequency-dependent effects of rPMS, whereby low-frequency stimulation primarily reduces spasticity and muscle stiffness, and high-frequency stimulation enhances motor recovery. Optimizing the rPMS parameters for specific clinical applications may further enhance its therapeutic potential in pain management and rehabilitation.

## 7. Combined Approach

**Figure 9:**
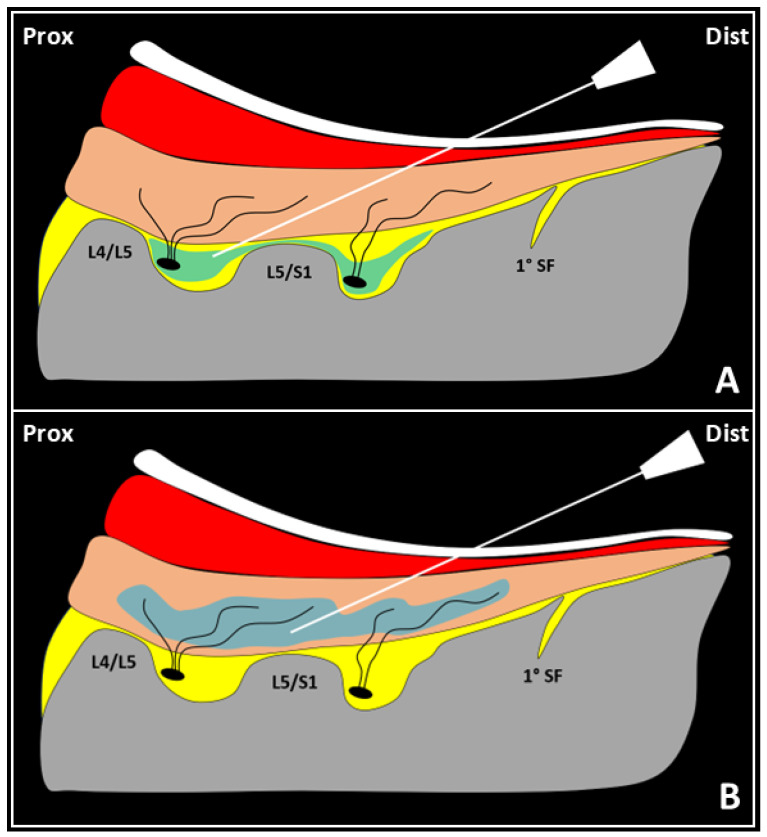
(**A**) The needle’s tip (white) is advanced using an in-plane, distal-to-proximal approach within the fat-filled interface (yellow) between the facet bone (grey) and multifidus muscle (orange) to infiltrate the medial branches (black dots) of the dorsal rami of the lumbar spinal nerves. (**B**) The mixture (light blue) is injected into the lumbar multifidus muscle to target both the intramuscular ramifications (black lines) of the medial branches and surrounding muscle fibers. Prox: proximal; Dist: distal; white band: superficial soft tissues; red band: other erector spinae muscle; L4/L5 and L5/S1: facets; 1° SF: first sacral foramen.

A pilot study by Wu et al. [31] demonstrated the effectiveness of integrating ultrasound-guided injections (USGIs) of a mixture containing 5 mL of 50% dextrose and 5 mL of 1% lidocaine into the multifidus muscle, along with 12 sessions of repetitive rPMS, for managing chronic low back pain. The injection proceeded as follows: during the first phase, using an in-plane technique and distal-to-proximal approach, the needle’s tip was advanced within the fat-filled histological interface between the cortical bone of the facets and the undersurface of the multifidus muscle, which also might have infiltrated the medial branches of the dorsal rami of the lumbar spinal nerves. Then, after retracting the needle, the mixture was injected into the lumbar multifidus muscle to target both the intramuscular ramifications of the medial branches of the dorsal rami of the lumbar spinal nerves and the surrounding muscle fibers (Figure 9).

**Figure 10:**
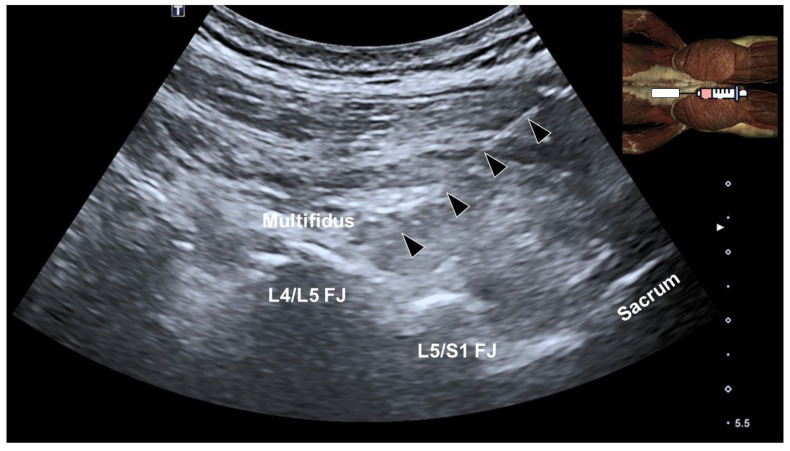
Ultrasound-guided injection into the lumbar facet joints and the overlying multifidus muscle in the sagittal plane. Arrowheads, needle trajectory; FJ, facet joint.

The study included three participants with chronic low back pain who received bilateral injections at the L4/L5 and L5/S1 facet joints, along with multifidus injections using a peppering technique in the sagittal plane (Figure 10 and Video S3). Following the injections, participants underwent 12 sessions of rPMS targeting the bilateral lumbosacral region. Pain intensity and disability were assessed using the visual analog scale and Oswestry Disability Index at baseline, and after 6 and 12 sessions. While subjects had substantial pain and functional impairment at baseline, by the end of 12 sessions, a significant improvement was observed both in terms of pain and disability. These findings suggest that coupling USGIs with rPMS may provide rapid and sustained symptom relief.

Several key advantages support this combined approach for managing low back pain. USGIs provide precise, localized pain relief and tissue healing, while rPMS stimulates neuromuscular recovery. This dual mechanism addresses both nociceptive and neuromuscular components of chronic low back pain, potentially yielding superior outcomes compared to either treatment alone. Additionally, rPMS sessions can be administered by trained therapists or assistants following USGIs, optimizing the clinical workflow and resource utilization. The integration of targeted injections with neuromodulatory stimulation facilitates faster functional improvement, as evidenced by the rapid decline in pain and disability scores over a relatively short treatment duration.

Despite these promising findings, several considerations remain to be elucidated. The success of USGIs depends on accurate needle placement, requiring specialized training in ultrasound interpretation and injection techniques. Similarly, rPMS parameters—including the frequency, intensity, and duration—must be carefully calibrated according to individual patient needs, necessitating further research to establish standardized protocols. Future studies should focus on validating the long-term efficacy of this integrated approach through larger randomized controlled trials. Additionally, they should investigate whether the benefits of this combination approach result from an additive or synergistic effect, using a multi-arm trial design to compare rPMS alone, multifidus injection alone, and the combination of rPMS and multifidus injection. Additionally, exploring the mechanistic interactions between USGIs and rPMS may provide further insights into optimizing treatment strategies for refractory low back pain. A combination of precision-guided injections and non-invasive neuromodulation represents a promising, forward-thinking strategy for chronic musculoskeletal pain, with the potential to reshape conventional treatment paradigms.

Finally, although USGI is a precise technique for targeting injection sites, its effectiveness depends on more than just the use of ultrasound [32]. The success of the procedure is influenced by two main factors: (1) the exact location of the injection and (2) the choice of injectate. Injections can be delivered into various structures, including muscles, joints, the epidural space, or peripheral nerves, with injectates such as dry needling, saline, lidocaine, steroids, or dextrose. Each injectate has unique therapeutic effects and mechanisms of action. Additionally, certain regions, such as the multifidus muscle, may be accurately targeted without ultrasound guidance, emphasizing the role of anatomical knowledge and clinical expertise in achieving effective outcomes. Furthermore, given that various regimens (e.g., steroids, dextrose) and different injection sites/targets (e.g., facet [33] and lumbar nerve root [34]) have shown some efficacy in treating lower back pain, the optimal algorithm for combining these regimens with rPMS remains to be explored in future studies.

## 8. Conclusions

Both USGIs and rPMS offer innovative, effective solutions for managing chronic painful conditions such as MPS and low back pain. By leveraging advancements in imaging and electromagnetic technologies, clinicians can deliver targeted, patient-centered care. The integration of the two modalities would not only enhance the treatment outcome but also optimize healthcare resources, paving the way for broader adoption in pain management strategies. Future studies should also explore the potential effects of active exercise in conjunction with these treatments, as it may further enhance patient outcomes and contribute to a more comprehensive approach to pain management.

## Data Availability

No new data were created or analyzed in this study. Data sharing is not applicable to this article.

## Supplementary Materials

The following supporting information can be downloaded at: https://www.mdpi.com/article/10.3390/life15040563/s1, Video S1: Ultrasound imaging of the hamstring muscle during TENS stimulation; Video S2: Ultrasound imaging of the hamstring muscle during rPMS stimulation; Video S3: Ultrasound guided multifidus injection.
